# Resistencia a la meticilina y producción de biopelícula en aislamientos clínicos de *Staphylococcus aureus* y *Staphylococcus* coagulasa negativa en México

**DOI:** 10.7705/biomedica.4131

**Published:** 2019-09-01

**Authors:** Ayerim García, Carlos Martínez, Rosa Isela Juárez, René Téllez, Marco Antonio Paredes, María del Rocío Herrera, Silvia Giono

**Affiliations:** 1 Laboratorio de Bacteriología Médica, Departamento de Microbiología, Escuela Nacional de Ciencias Biológicas, Instituto Politécnico Nacional, Ciudad de México, México Instituto Politécnico Nacional Departamento de Microbiología Escuela Nacional de Ciencias Biológicas Instituto Politécnico Nacional Ciudad de México Mexico; 2 Servicio de Bioquímica, Instituto Nacional de Rehabilitación “Luis Guillermo Ibarra Ibarra”, Ciudad de México, México Servicio de Bioquímica Instituto Nacional de Rehabilitación “Luis Guillermo Ibarra Ibarra” Ciudad de México México; 3 Laboratorio Central de Patología Clínica, Instituto Nacional de Rehabilitación “Luis Guillermo Ibarra Ibarra”, Ciudad de México, México Laboratorio Central de Patología Clínica Instituto Nacional de Rehabilitación “Luis Guillermo Ibarra Ibarra” Ciudad de México México; 4 Laboratorio de Neurobiología, Instituto Nacional de Rehabilitación “Luis Guillermo Ibarra Ibarra”, Ciudad de México, México Laboratorio de Neurobiología Instituto Nacional de Rehabilitación “Luis Guillermo Ibarra Ibarra” Ciudad de México México

**Keywords:** *Staphylococcus aureus* resistente a meticilina, biopelículas, México., Methicillin-resistant *Staphylococcus aureus*, biofilms, México.

## Abstract

**Introducción.:**

Las infecciones por *Staphylococcus aureus* y *Staphylococcus* coagulasa negativa multirresistentes a los antibióticos y asociadas con la atención en salud tienen un gran impacto epidemiológico por su alta morbimortalidad; además, se han relacionado con la formación de biopelículas, lo cual también se asocia con la resistencia a los antimicrobianos.

**Objetivo.:**

Determinar la resistencia a la meticilina y cuantificar la producción de biopelículas para establecer su posible relación con los aislamientos clínicos de *S. aureus* y *Staphylococcus* coagulasa negativa.

**Materiales y métodos.:**

Se estudiaron 11 cepas de *S. aureus* y 12 de *Staphylococcus* coagulasa negativa. La resistencia a la meticilina se determinó con discos de cefoxitina tomando como valores de referencia los estándares del *Clinical Laboratory Standards Institute* (CLSI) de 2018. La producción de biopelícula se cuantificó con cristal violeta. Los genes *mecA* e *icaADBC* se identificaron mediante reacción en cadena de la polimerasa (PCR), y se hizo un análisis bivariado con la prueba de ji al cuadrado y el coeficiente V de Cramér, utilizando el programa SPSS™, versión 20.0.

**Resultados.:**

Nueve cepas de *S. aureus* fueron resistentes a la meticilina (SARM) y dos fueron sensibles. Ocho cepas de *Staphylococcus* coagulasa negativa fueron resistentes y cuatro fueron sensibles. El genotipo *mecA* se encontró en ocho de las nueve cepas de *S. aureus* y en seis de las ocho de *Staphylococcus* coagulasa negativa resistentes a meticilina. Todas las cepas formaron biopelícula. Diez cepas de *S. aureus* y 11 de *Staphylococcus* coagulasa negativa presentaron el genotipo *icaADCB.* No se encontró asociación entre la resistencia a meticilina y la formación de biopelícula.

**Conclusiones.:**

La cefoxitina es suficiente para determinar el fenotipo resistente a meticilina y se asoció con el genotipo *mecA*. Las cepas resistentes a la meticilina y poseedoras del gen *mecA* pueden presentar un mecanismo de resistencia alterno. Los dos grupos de cepas formadoras de biopelícula se relacionaron con la presencia del operón *icaADCB.* La formación de biopelícula y la resistencia a la meticilina se expresaron como características independientes en los dos grupos de cepas.

## Abstract (en)

**Introduction:**

Infections associated with health care caused by *S. aureus* and coagulase- negative *Staphylococci* multi-resistant to antibiotics cause a high epidemiological impact due to their high morbidity and mortality. Biofilm formation, which has been associated with antimicrobial resistance, can also occur.

**Objectives:**

To determine methicillin resistance and to quantify the biofilm production to establish if there is a relationship in clinical isolates of *S. aureus* and coagulase-negative *Staphylococci*.

**Material and methods:**

A total of 11 strains of *S. aureus* and 12 of coagulase-negative *Staphylococci* were studied. Methicillin resistance was determined with cefoxitin discs and the *Clinical Laboratory Standards Institute* (CSLI), 2018 reference values. Biofilm production was quantified by the crystal violet method. The *mecA* and *icaADBC* genes were identified by PCR. A bivariate analysis was performed with chi-square (c2) and Cramér’s V statistical tests, using SPSS™, version 20.0 software.

**Results:**

Nine *S. aureus* strains were methicillin-resistant and two were sensitive. Eight coagulase-negative *Staphylococci* strains were resistant and four were sensitive. The *mecA* genotype was found in eight of the nine *S. aureus* resistant strains and six of eight resistant coagulase-negative *Staphylococci*. All strains formed biofilms. Ten strains of *S. aureus* and 11 of coagulase-negative *Staphylococci* presented the *icaADCB* genotype. No association was found between methicillin-resistance and biofilm formation.

**Conclusions:**

Cefoxitin is enough to define the resistance phenotype and is associated with the *mecA* genotype. All strains formed biofilms and were related to the presence of the *icaADCB* operon. Biofilm formation and methicillin resistance were independent features in both groups of strains.

Las infecciones asociadas con la atención en salud representan un problema de salud pública debido a su alta morbilidad y mortalidad. Los agentes patógenos que las causan en México pertenecen al grupo de bacterias Gram positivas *Staphylococcus aureus* y *Staphylococcus* coagulasa negativa, al de las bacterias Gram negativas *Acinetobacter baumannii* y *Pseudomonas aeruginosa*, y al de especies pertenecientes a Enterobacteriaceae ^1^^,^^2^.

*Staphylococcus* spp. son cocos Gram positivos de 0,5 a 1,5 μm de diámetro que se agrupan en racimos, son inmóviles, anaerobios facultativos, fermentadores de glucosa, positivos para catalasa y negativos para oxidasa, con un contenido de G+C de 30 a 39 %, y son oportunistas ^3^. *Staphylococcus aureus* es la especie más virulenta; se la considera una bacteria extracelular que induce alteraciones en los tejidos y produce lesiones localizadas con supuración y cicatrización, y es responsable de varias infecciones en el humano ^4^.

Los factores que incrementan la probabilidad de adquirir dichas infecciones incluyen la hospitalización durante periodos prolongados, los procedimientos preoperatorios, la utilización de catéteres o prótesis y la permanencia en lugares de alto riesgo (unidades de cuidados intensivos, entre otros), pero los principales factores de riesgo son la virulencia, el potencial para formar abscesos y la multirresistencia a los antibióticos ^5^.

La Red Hospitalaria de Vigilancia Epidemiológica de México ha reportado que la mortalidad en pacientes infectados con *S. aureus* varía de 5 a 70 %. La información proveniente de los hospitales generales, pediátricos, universitarios y de especialidades señala que *S. aureus* ocupó el tercer lugar en morbilidad y el cuarto lugar en mortalidad en el periodo de 1997 a 2003 ^5^.

La resistencia del género *Staphylococcus* a los β-lactámicos, como las penicilinas, las cefalosporinas y los carbapenémicos, es un problema de salud pública ^6^. La resistencia a la penicilina en *S. aureus* surgió a finales de la década de 1950 y obligó al desarrollo de nuevos antimicrobianos. Así aparecieron las primeras cefalosporinas estables frente a las penicilinasas y las penicilinas semisintéticas, entre ellas la meticilina. Un año después de su introducción, se aisló en Europa la primera cepa de *S. aureus* resistente a la meticilina (SARM) y, en 1963, se reportó el primer brote hospitalario; desde entonces se han notificado en todo el mundo ^4^.

Los mecanismos de resistencia de *S. aureus* a los β-lactámicos son la producción de enzimas β-lactamasas, la presencia de proteínas ligadas a la penicilina (*Penicillin Binding Protein*, PBP) modificadas (conocida como resistencia intrínseca a la meticilina) y los fenómenos de tolerancia. Las penicilinas resistentes a penicilinasas (oxacilina, meticilina, cloxacilina) poseen una estructura molecular que las protege frente a la acción de las β-lactamasas. El mecanismo de resistencia de *S. aureus* a la meticilina se basa en la síntesis de una nueva PBP (PBP2a o PBP2´), la cual exhibe poca afinidad por la meticilina y otros β-lactámicos, bloquea la llegada del antibiótico a su sitio blanco y produce, así, un patrón de resistencia ^7^.

El elemento genético cromosómico responsable de la resistencia es el gen *mecA,* cuya expresión depende de dos genes, el *mecR1,* que regula la transcripción, y el *mecI,* que codifica la proteína represora. El antibiótico β-lactámico induce un proceso catalítico en la membrana bacteriana, y el gen *mecA* se transcribe y se sintetiza en la proteína de membrana PBP2a ^8^^,^^9^.

Otras modalidades de resistencia en las que no se evidencia la presencia del gen *mecA*, son la resistencia límite a la oxacilina (*Borderline Oxacillin-Resistant S. aureus,* BORSA) en bacterias hiperproductoras de β-lactamasas, y la resistencia modificada (*Modified S. aureus,* MODSA) en aquellas que presentan modificaciones en la afinidad de las PBP 1, 3 y 4, por lo que exhiben débil resistencia a la meticilina. Otros genes de resistencia son el gen *blaZ* y el *fem* (factor esencial de resistencia a la meticilina) ^7^.

Asimismo, está la producción de biopelículas, la cual se considera un factor de virulencia. La biopelícula es una comunidad de microorganismos recubiertos de un polímero extracelular o matriz de exopolisacáridos, con la capacidad de adherirse a superficies bióticas o abióticas ^10^; se ha demostrado que son estructuras tridimensionales ^11^. La matriz de exopolisacáridos favorece el intercambio de metabolitos con el exterior y confiere una barrera protectora contra ambientes adversos, como la hiperosmolaridad, la anaerobiosis, los anticuerpos, los macrófagos y los antibióticos. Este crecimiento ‘protegido’ permite la supervivencia en un medio antagonista ^11^^).^

La adhesina intercelular de polisacáridos (*Polysaccharide Intercellular Adhesin*, PIA), llamada poli-N-acetilglucosamina (PNAG), es un polímero de 28 kDa, homoglucano lineal de la glucosamina, con una estructura bioquímica de β-1,6-N-acetil-glucosamina ^10^. La biosíntesis de la PIA de *Staphylococcus* spp. participa en la formación de biopelículas ^10^^,^^12^ y se sintetiza por la acción de cuatro proteínas homólogas organizadas en el operón *ica,* las cuales forman parte del grupo de adhesinas intercelulares (*Intercellular Cluster Adhesin,* ICA) codificadas por los genes *icaA*, *icaD*, *icaB* e *icaC*^13^. El gen *bap* puede mediar un mecanismo independiente de la PIA para la formación de la biopelícula ^13^^,^^14^. Por otra parte, la proteína Bap (*Biofilm-Associated Protein*) se localiza en la superficie bacteriana asociada a la pared celular y desempeña un papel relevante en los procesos infecciosos de la mastitis bovina ocasionada por diferentes especies de *Staphylococcus*^13^^,^^15^.

Existe un interés creciente en la formación de biopelículas de bacterias patógenas capaces de adherirse a dispositivos como prótesis ortopédicas, válvulas cardiacas artificiales, marcapasos, injertos de plástico y dispositivos intravenosos temporales o permanentes ^11^. La tolerancia a los agentes antimicrobianos en el 60 % de las infecciones bacterianas está asociada a la formación de biopelículas y la concentración del agente antimicrobiano requerida para introducirse en la biopelícula y tener efecto en las bacterias ^16^.

Las cepas de SARM se asocian con infecciones hospitalarias en las que se se pueden formar biopelículas, y se caracterizan por ser persistentes, virulentas y difíciles de eliminar ^10^. En ese contexto, el objetivo del estudio fue investigar la asociación de la resistencia a la meticilina de los genotipos *mecA* e *icaADCB* con la formación de biopelículas en cepas de *S. aureus* y *Staphylococcus* coagulasa negativa, aisladas de muestras clínicas de pacientes hospitalizados en el Instituto Nacional de Rehabilitación “Luis Guillermo Ibarra Ibarra” de Ciudad de México.

## Materiales y métodos

### Cepas bacterianas

Las cepas utilizadas provenían de muestras clínicas (heridas quirúrgicas, secreciones bronquiales, hemocultivos, puntas de catéter, cultivos óseos) recolectadas durante el periodo de febrero a julio del 2010. Se estudiaron 23 cepas de *Staphylococcus* spp.: 11 de *S. aureus* y 12 de *Staphylococcus* coagulasa negativa. Los aislamientos se obtuvieron por resiembra en placas de gelosa con sangre de carnero y medio agar de sal y manitol. Todas las cepas aisladas fueron positivas para catalasa y negativas para oxidasa. Según la prueba de coagulasa, las cepas se clasificaron como *Staphylococcus* coagulasa positivas, entre ellas las de *S. aureus*, y *Staphylococcus* coagulasa negativas. La identificación del género y de la especie se llevó a cabo con un panel bioquímico usando un sistema automatizado (MicroScan Instruments, Dade-Behring). Las cepas se conservaron en caldo de cerebro y corazón con glicerol al 20 % y a -70 °C hasta su procesamiento.

Se usó la cepa ATCC 25923 de *S. aureus* como control negativo para las pruebas de resistencia a la meticilina, la ATCC 43300, como control positivo de la presencia del gen *mecA*, la ATCC 27543, como control positivo para la producción de biopelícula, y la ATCC 12228, para la detección de los genes *icaA*, *icaD*, *icaB* e *icaC.*

### Prueba de difusión en disco para oxacilina y cefoxitina

La sensibilidad a la meticilina se determinó mediante el método de Kirby y Bauer o de difusión mediante el uso de discos de cefoxitina (30 μg) (BD BBL Sensi-Disc Antimicrobial™) como método de referencia, según las guías y normas del CLSI (2018) para establecer la sensibilidad a los antimicrobianos mediante discos ^17^.

Para preparar el inóculo, se seleccionaron cuatro colonias que se colocaron en tubos con 4 ml de caldo Müeller-Hinton; se ajustó la turbidez al 0,5 usando un nefelómetro con el estándar de McFarland (1,5 x 108 UFC/ml); se sembraron con hisopo en tres direcciones en placas de agar Müeller- Hinton y se colocaron los discos.

Las placas se incubaron durante 24 horas a 37 °C y se midieron los diámetros de los halos de inhibición en milímetros usando un escalímetro; los resultados se compararon con los valores establecidos en el manual del CLSI (2018) para su clasificación como resistentes o sensibles.

### Producción de biopelícula

La producción de biopelícula se cuantificó con el método de titulación en microplaca de poliestireno utilizando cristal violeta ^18^. A partir de un cultivo de 24 horas de cada cepa, se preparó una suspensión en caldo de cultivo Müeller-Hinton, y se ajustó a la turbidez del tubo número 3 de McFarland, equivalente a 9,0 x 108 UFC/ml. Cada ensayo se hizo por cuadruplicado para cada control y para las cepas problemáticas.

Se agregaron 100 μl de esa suspensión a cuatro pozos de microplacas de polipropileno de 96 pozos con fondo plano (Nunc MicroWell™); en los pozos de control negativo se agregó solo el medio Müeller-Hinton y, después, se incubó durante 24 horas a 37 °C. Se aspiró el contenido con micropipetas empleando puntas de 200 μl estériles y los pozos se lavaron con 100 μl de solución tampón fosfato salino (PBS) estéril.

Las bacterias adheridas se fijaron con 100 μl de glutaraldehído al 2,5 % durante un minuto a temperatura ambiente, se retiró el exceso aspirando nuevamente y cada uno de los pozos se lavó con 100 μl de PBS. Se agregaron 100 μL de cristal violeta al 4 % en cada pozo y se incubó a temperatura ambiente durante dos minutos; se retiró el exceso de cristal violeta por aspiración y se lavaron dos veces con PBS. El cristal violeta adherido en cada pozo se disolvió con 100 μl de alcohol y acetona en proporción 80:20 (v/v), y el volumen final se ajustó a 2 ml con la misma solución.

Las lecturas se hicieron con un espectrofotómetro a una absorbancia de 570 nm: se promediaron los valores de las cuatro lecturas de absorbancia, y se calcularon la media y la desviación estándar (DE) para cada una de las cepas y el control negativo. El punto de corte (AbsC) se definió como el valor de tres DE por encima del promedio de las absorbancia del control negativo (AbsC=absorbancia media de los controles + 3X DE de los controles). La absorbancia final de cada una de las cepas se calculó sustrayendo el valor de AbsC (absorbancia=absorbancia de una cepa - AbsC).

Cuando el valor obtenido de absorbancia resultó negativo, se consideró como valor cero; cualquier valor positivo indicaba que había producción de biopelícula. La producción de biopelículas se agrupó en cuatro categorías: nula o no adherente (absorbancia≤0,001), baja adherencia (absorbancia=0,001-0,500), moderada (absorbancia=0,051-0,900) y alta adherencia (absorbancia≥ 0,901) ^19^^,^^20^.

### Extracción del ADN genómico

Para la extracción del ADN genómico, se empleó el método de tiocianato de guanidina: cada cepa se sembró por estría masiva en agar Müeller-Hinton y se incubó a 37 °C durante 24 horas; las colonias se suspendieron en un ml de NaCl al 0,85 %, se centrifugaron a 14,000*g* durante cinco minutos, se lavaron dos veces con 200 μl de agar de *Salmonella* y *Shigella* y se suspendieron de nuevo en 100 μl de solución tampón tris EDTA (1mM EDTA, 10 mM Tris-HCl, pH 8,0).

Se agregaron después 500 μl de solución tampón de tiocianato de guanidinio 5M y EDTA (0,1M pH 6,0), y 25 μl de N-Lauril-sarcocinato de sodio al 10 %; se mezcló por inversión durante cinco minutos a temperatura ambiente y cinco minutos en hielo. Se agregaron 250 μl de acetato de amonio 7,5M y 500 μl de cloroformo alcohol isoamílico en proporción 24:1 (v/v); se mezcló por inversión y se centrifugó a 14.000*g* durante siete minutos. Se recuperó la fase acuosa, se agregaron 500 μl de isopropanol frío y se dejó a -20 °C toda la noche.

El ADN se recuperó por centrifugación durante 10 minutos a 10.000*g*; la pastilla obtenida se lavó con 200 μl de etanol frío al 70 % (v/v), se centrifugó a 10.000*g* y se dejó secar al aire durante dos horas para, finalmente, disolverla en 80 μl de agua para biología molecular.

### Identificación de los genes mecA e icaADBC por reacción en cadena de la polimerasa (PCR)

Se emplearon los iniciadores específicos diseñados para la amplificación de los genes *mecA* e *icaADBC* (21). Las secuencias de los iniciadores específicos, las condiciones de amplificación y los tamaños esperados de cada amplicón se presentan en el cuadro 1.

**Cuadro 1:** Secuencias de los iniciadores específicos y condiciones para la amplificación de los genes *mecA* e *icaADBC*

Iniciador	Secuencia	Condiciones de alineamiento	Amplicón (pb)
icaA-F	5´-CGTTGATCAAGATGCACC-3´	30s/50 °C	319 pb
icaA-R	5´-CCGCTTGCCATGTGTTG-3´	30s/50 °C	
icaB-F	5´-TGGATTAACTTTGATGATATGG-3´	60s/54 °C	409 pb
icaB-R	5´-AGGAAAAAGCTGTCACACC-3´	60s/54 °C	
icaC-F	5´-GGTCAATGGTATGGCTATTT-3´	60s/54 °C	148 pb
icaC-R	5´-CGAACAACACAGCGTTTC-3´	60s/54 °C	
icaD-F	5´-GGTCAAGCCCAGACAGAG-3´	60s/54 °C	150 pb
icaD-R	5´-GAAATTCATGACGAAAGTATC-3´	60s/54 °C	
mecA-F	5´- TGGCTATCGTGTCACAATCG-3´	30s/55 °C	310 pb
mecA-R	5´-CTGGAACTTGTTGAGCAGAG-3´	30s/55 °C	

El volumen final de la mezcla de reacción para la amplificación por PCR fue de 25 μl; las concentraciones finales de cada uno de los iniciadores fueron de 0,1 μM, 1,5 mM de MgCl2, 2,5 mM de dNTP y agua para biología molecular.

Se utilizaron 30 ciclos de desnaturalización inicial durante cinco minutos a 94 °C, desnaturalización durante 30 s a 94 °C, alineamiento y extensión durante 1,5 minutos a 72 °C y una extensión final de 10 minutos a 72 °C.

Los productos de la amplificación se visualizaron mediante electroforesis en agarosa al 1,5 % en solución tampón TBE (tris, borato, EDTA) teñido con bromuro de etidio; el tamaño de los amplicones se calculó comparándolos con un marcador de talla molecular comercial de 100 pb. El control positivo para la presencia del gen *mecA* fue el ADN de la cepa ATCC43300 de *S. aureus.*

### Análisis estadístico

El análisis estadístico de la producción de biopelícula se hizo con el programa SPSS™, versión 20.0. Se hizo un análisis bivariado con las pruebas de ji al cuadrado y el coeficiente V de Cramér para examinar la asociación entre la resistencia a la meticilina y la formación de biopelícula.

## Resultados

Las 23 cepas de *Staphylococcus* spp. de origen clínico aisladas se dividieron en dos grupos: el primero con 11 (48 %) cepas de *S. aureus*, y el segundo con 12 (52 %) cepas de *Staphylococcus* coagulasa negativa.

El fenotipo resistente a meticilina se encontró en 9 (81,8 %) cepas de *S. aureus* y 2 (18,2 %) fueron sensibles. En las cepas del grupo *Staphylococcus* coagulasa negativa, 8 (66,7 %) fueron resistentes y, 4 (33,3 %), sensibles a meticilina (cuadro 2).

**Cuadro 2:** Fenotipos de sensibilidad y resistencia a la meticilina establecidos por difusión en disco de las cepas de los grupos de *Staphylococcus aureus* y *Staphylococcus aureus* coagulasa negativa

Grupo de cepas	Fenotipo de sensibilidad a meticilina		Total n (%)
	Sensible a cefoxitina n (%)	Resistente a cefoxitina n (%)	
Staphylococcus aureus	2 (9)	9 (39)	11 (48)
*Staphylococcus aureus* coagulasa negativa	4 (17)	8 (35)	12 (52)
Total	6 (26)	17 (74)	23 (100)

**Figura 1:**
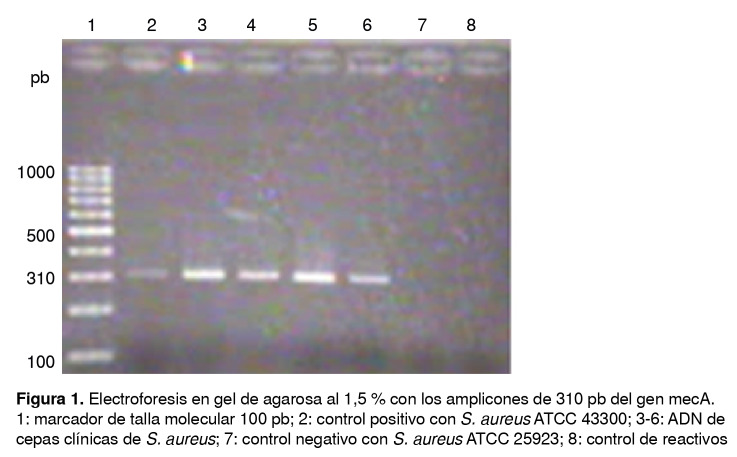
Electroforesis en gel de agarosa al 1,5 % con los amplicones de 310 pb del gen mecA. 1: marcador de talla molecular 100 pb; 2: control positivo con *S. aureus* ATCC 43300; 3-6: ADN de cepas clínicas de *S. aureus*; 7: control negativo con *S. aureus* ATCC 25923; 8: control de reactivos

En cuanto a la correlación entre el fenotipo de resistencia a la meticilina y la presencia del gen *mecA*, el producto de amplificación mediante PCR de punto final fue el esperado, un amplicón de 310 pares de bases observado por electroforesis en gel de agarosa al 1,5 % (figura 1); de las 23 cepas, 18 (78 %) presentaron este gen*.*

De las nueve cepas de SARM, ocho presentaron el gen *mecA* y solo en una no se detectó. En diez cepas de *Staphylococcus* coagulasa negativa se detectó el gen *mecA*, es decir, se encontró en las ocho cepas resistentes a la meticilina y, además, en dos cepas con fenotipo sensible a la meticilina y presencia del gen *mecA*^19^.

En cuanto a la formación de biopelícula como mecanismo de virulencia, se encontró que con las categorías establecidas: nula o no adherente (absorbancia≤0,001), de baja adherencia (absorbancia=0,001-0,500), de moderada adherencia (absorbancia=0,051-0,900) y de alta adherencia (absorbancia≥0,901), todas las cepas produjeron biopelícula cuando se empleó la cepa ATCC 27543 como control positivo para dicha producción (cuadro 3).

**Cuadro 3:** Capacidad de producción de biopelícula en cepas de los grupos de *Staphylococcus aureus* y *Staphylococcus aureus* coagulasa negativa

**Grupo de cepas**	Fenotipo de producción de biopelícula			
	Baja n (%)	Moderada n (%)	Alta n (%)	Total n (%)
*Staphylococcus aureus*	3 (13)	7 (30)	1 (5)	11 (52)
*Staphylococcus aureus coagulasa negativa*	2 (9)	7 (30)	3 (13)	12 (48)

En el grupo de cepas de *Staphylococcus* coagulasa negativa se encontraron tres (13 %) con alta producción en comparación con una (5 %) de *S. aureus.* En ambos grupos hubo el mismo número de cepas (7; 30 %) con producción moderada de biopelícula, en tanto que solo tres cepas de *S. aureus* y dos de *Staphylococcus* coagulasa negativa presentaron el fenotipo de baja producción de biopelícula.

**Figura 2:**
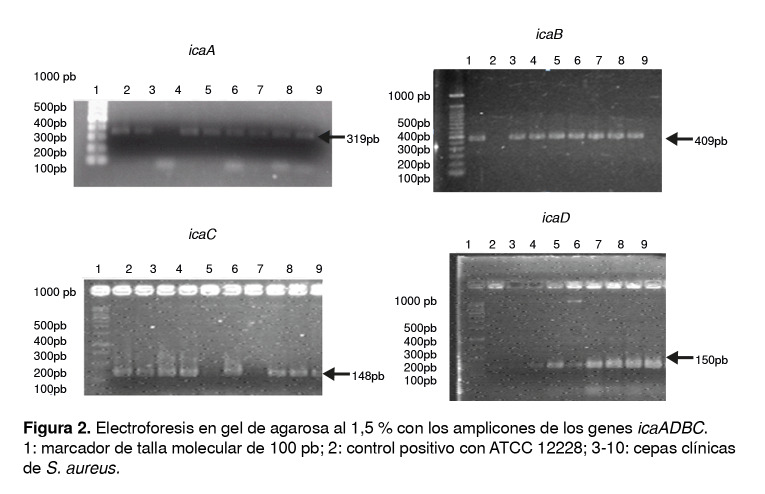
Electroforesis en gel de agarosa al 1,5 % con los amplicones de los genes *icaADBC*. 1: marcador de talla molecular de 100 pb; 2: control positivo con ATCC 12228; 3-10: cepas clínicas de *S. aureus.*

Se amplificaron de manera independiente los genes *A*, *D*, *B* y *C* del operón *ica*, con el propósito de correlacionar su presencia con el fenotipo de formación de biopelícula en las cepas de *Staphylococcus* spp*.* Las cepas de los dos grupos presentaron el operón *icaADBC* al usar como control la cepa ATCC 12228 de *S. epidermidis* para la presencia de los genes *icaA*, *icaD*, *icaB* e *icaC* (figura 2). La electroforesis en geles de agarosa al 1,5 % evidenció en cada cepa los amplicones de cada uno de los genes con el tamaño esperado.

## Discusión

En el análisis estadístico de asociación entre la producción de biopelícula y los dos grupos de cepas de *S. aureus* y *Staphylococcus* coagulasa negativa mediante el V de Cramér y la prueba de ji al cuadrado, se encontraron valores de V de 0,245 y de p de 0,111, lo que indica que las dos variables fueron independientes entre sí. La producción de biopelícula se asoció con las cepas SARM *Staphylococcus* coagulasa negativa resistente a la meticilina (*Methicillin-Resistant Coagulase-Negative Staphylococcus aureus*, MRSCN). El análisis estadístico también determinó que la producción de biopelícula se daba independientemente de la resistencia a la meticilina, con un valor de V de 0,115 y uno de p de 0,252. 

Las infecciones asociadas con la atención en salud constituyen un grave problema de salud pública en todo el mundo y afectan gravemente a los países de menores recursos. Las infecciones hospitalarias se encuentran entre las principales causas de morbilidad y mortalidad, y su manejo ocasiona costos elevados para los pacientes y el sistema de salud ^22^.

*Staphylococcus aureus* y varias especies de *Staphylococcus* coagulasa negativa, se caracterizan por ser resistentes a la meticilina, lo que, a su vez, les confiere resistencia a varios antibióticos β-lactámicos y dificulta enormemente su erradicación. Por ello, ha sido necesario establecer protocolos específicos de tratamiento con otros antibióticos para las infecciones causadas por este tipo de *Staphylococcus* spp*.*, con la consiguiente presión selectiva que favorece la diseminación de dichas cepas.

La acumulación y la diseminación de la resistencia en *S. aureus* son producto del intercambio de factores determinantes de resistencia preexistentes en elementos genéticos móviles, como los plásmidos y los transposones ^5^.

En las muestras de *Staphylococcus* spp. analizadas en este estudio fue posible determinar el fenotipo resistente a la meticilina utilizando discos con cefoxitina. Las cepas SARM presentaron el mismo rango de sensibilidad y especificidad frente a los dos antibióticos. Estos resultados coinciden con un estudio comparativo de varios métodos de detección de cepas aisladas de muestras clínicas hospitalarias, que presentaron una sensibilidad de 98 % y una especificidad de 100 % utilizando discos de cefoxitina ^23^.

En las cepas de *Staphylococcus* coagulasa negativa se detectó el fenotipo de resistencia a la meticilina con los valores de corte de los halos de inhibición. En este sentido, los resultados del presente estudio son similares a los hallados por otros autores ^24^.

Los métodos de tipificación molecular basados en la detección del gen *mecA* mediante PCR de punto final están cada vez más al alcance de los laboratorios clínicos y representan una alternativa rápida y eficaz para la detección de cepas SARM. Además, tienen la gran ventaja de no depender de condiciones de cultivo especiales y se pueden usar en un gran número de aislamientos para establecer el fenotipo de resistencia a la meticilina y sus asociaciones.

En el presente estudio encontramos una correlación entre el fenotipo y el genotipo de resistencia a la meticilina; únicamente en una cepa de fenotipo SARM no se encontró el gen *mecA.*

En cuanto a la formación de biopelícula como factor de virulencia de los estafilococos mediante su adherencia y colonización de células, tejidos y materiales inertes, su determinación *in vitro* en cepas clínicas ha evolucionado e incluye desde métodos poco reproducibles, como el agar de rojo Congo, hasta los métodos espectrofotométricos semicuantitativos sobre superficies inertes ^25^.

El método empleado en el presente trabajo presentó una buena reproducibilidad y permitió establecer en los dos grupos tres niveles de formación de biopelícula, predominantemente el moderado.

En diversos estudios se ha demostrado que tanto *S. aureus* como *Staphylococcus* coagulasa negativa de origen clínico tienen la misma capacidad de formar biopelículas ^26^. En cepas clínicas de *S. aureus*, resistentes y sensibles a meticilina se ha detectado un nivel moderado de formación de biopelículas, en menor medida, un nivel alto, y en muy pocas cepas, un nivel bajo, sin que se pudiera establecer una correlación entre la resistencia a la meticilina y la capacidad de producción de biopelícula.

Las cepas de *S. aureus* aisladas de la piel de pacientes presentaron una mayor capacidad de producir biopelícula, hecho que favorecería las condiciones para la colonización y la persistencia en el humano si se considera que se trata de uno de los principales microorganismos de la microbiota de la piel ^27^^).^

El operón *ica* es un elemento importante en la formación y acumulación de biopelícula; consta de cuatro genes que codifican para la adhesina intercelular de polisacárido de las bacterias. En este estudio se encontró que el 91 % de las cepas del grupo de *S. aureus* y el 92 % de las del grupo de *Staphylococcus* coagulasa negativa presentaron los genes *icaADBC*. La capacidad de formar biopelícula depende, en parte, de la actividad del locus *icaADBC* y del gen *icaR* implicados en la producción de la adhesina intercelular de polisacárido la cual es necesaria funcionalmente para la adherencia entre células y para la acumulación de biopelícula. Estos resultados coinciden con los de otro estudio ^14^, en el que se obtuvo una alta frecuencia de genes *icaADBC* en todos los aislamientos clínicos.

Aunque esta adhesina es un factor muy importante en la formación de biopelícula, se identificaron cepas que no codificaron para los genes *icaADBC* y, por lo tanto, no sintetizan el polisacárido**.** Este resultado no implica que no tengan la capacidad de formar biopelícula, sino que otros genes no incluidos en el estudio citado, como el *SigB*, el *SarA* y el *LuxS,* que también participan en la producción de biopelícula, podrían estar implicados ^14^.

La resistencia a la meticilina y la producción de biopelícula en los aislamientos clínicos de *S. aureus* y *Staphylococcus* coagulasa negativa son factores de virulencia que se expresan de manera independiente. Se obtuvo una importante correlación entre el fenotipo resistente a la meticilina y el genotipo del *mecA* en las cepas de origen clínico. Prácticamente todas las cepas fueron capaces de formar biopelícula, ya fuera por la participación del operón *ica* o por la de otros genes que no fueron parte de esta investigación.

Los estudios biotecnológicos fenotípicos y biomoleculares mediante PCR en infecciones hospitalarias han contribuido a un mejor conocimiento de su persistencia y resistencia antimicrobiana, y favorecen un mejor manejo clínico y terapéutico. La metodología empleada permitió aislar y clasificar las 23 cepas de *Staphylococcus* spp. procedentes de diferentes casos clínicos en dos grupos: *S. aureus* y *Staphylococcus* coagulasa negativa. Se pudo determinar mediante PCR que 18 de las 23 cepas presentaron el gen *mecA*, pero en una cepa con fenotipo de resistencia no se lo detectó, lo que sugiere un mecanismo de resistencia alterno. No se demostró asociación entre la producción de biopelícula y la resistencia a la meticilina.
